# Revision Carpal Tunnel Release With Umbilical Cord Allograft: A Four-Year Retrospective Cohort Study

**DOI:** 10.31486/toj.22.0079

**Published:** 2023

**Authors:** Nicholas L. Newcomb, Michael Nammour, Bhumit Desai, Loy Vaughan, Leslie E. Sisco-Wise

**Affiliations:** ^1^ Department of Orthopedic Surgery, University of New Mexico, Albuquerque, NM; ^2^ Department of Orthopedic Surgery, Ochsner Clinic Foundation, New Orleans, LA; ^3^ The University of Queensland Medical School, Ochsner Clinical School, New Orleans, LA

**Keywords:** *Allografts*, *carpal tunnel syndrome*, *median nerve*, *median neuropathy*, *reoperation*, *umbilical cord*

## Abstract

**Background:** Refractory symptoms of carpal tunnel syndrome can persist or reoccur after carpal tunnel release (CTR) surgery in 1% to 25% of patients, with up to 12% of patients requiring secondary surgery. If revision surgery is required, the results are much less successful compared to primary surgery. In this study, we investigated whether cryopreserved human umbilical cord allograft placement during CTR revision surgery improved short- and long-term surgical outcomes.

**Methods:** We conducted a single-center cohort analysis of patients between January 2015 and July 2018 who underwent secondary open revision CTR with umbilical cord allograft for recurrent or persistent compression neuropathy of the median nerve. Surgical outcomes of patients in the study group—reduction of pain, paresthesia, and weakness; complications; and Disabilities of the Arm, Shoulder and Hand (QuickDASH) scores—were compared to the outcomes of controls without umbilical cord allograft use who were operated on by the same surgeon between December 2011 and September 2015.

**Results:** A total of 37 patients underwent CTR with (n=26) and without (n=11) umbilical cord allograft (mean follow-up of 4 years). Following surgery, preoperative symptoms of pain (96% vs 73%, *P*=0.048) and paresthesia (100% vs 73%, *P*=0.014) were significantly improved in the patients who received umbilical cord allograft. Mean QuickDASH scores (19.0 vs 23.7, *P*=0.58) and preoperative weakness (90% vs 67%, *P*=0.14) were improved in the patients who received umbilical cord allograft but were nonsignificant. Short- and long-term complications were similar between groups (*P*=0.56, *P*=0.51, respectively).

**Conclusion:** This study suggests that human umbilical cord allograft placement during open revision CTR is safe and effective for improving long-term symptoms of compressive neuropathy in patients with recurrent carpal tunnel syndrome.

## INTRODUCTION

Carpal tunnel syndrome is the most common compressive neuropathy in the United States, affecting approximately 1% to 5% of the general population.^1^ Symptoms of carpal tunnel syndrome include pain, paresthesia, and weakness in the wrist and hand that can radiate to the forearm.^2^ When conservative treatment fails to relieve persistent symptoms, carpal tunnel release (CTR) is often performed to surgically release pressure on the median nerve. CTR is the most common hand and wrist surgery in the United States, with the number of procedures growing from 360,000 in 1996 to 577,000 in 2006.^3^ Despite the high success rate of CTR, symptoms can persist or reoccur in 1% to 25% of patients, with approximately 5% to 12% of patients requiring secondary surgery.^4,5^

Persistent symptoms are usually caused by incomplete release of the transverse carpal ligament. One hypothesis for recurrence of symptoms is excessive fibrous proliferation and scarring around the median nerve at the site of the initial decompression.^6^ This scarring can result in compression on the nerve, impairment of epineural blood flow leading to ischemia, and restriction in nerve gliding.^7^ Treatment of recurrent compressive neuropathy is difficult, with revision surgery having lower success rates and less predictable outcomes. Patients undergoing revision nerve decompression often have identical symptoms afterwards.^8^

To achieve better postsurgical outcomes, wrapping the median nerve with biomaterials (such as collagen or submucosa extracellular matrix) or using local flaps (such as muscle, fascia, or fat) has been attempted with variable outcomes.^8^ Nerve wrapping with cryopreserved amniotic membrane and umbilical cord is a potential technique that has not been well studied. Amniotic membrane has been shown to significantly decrease scar formation when used as a wrap around the ulnar^9^ and sciatic nerves^10^ in animal models. Other animal studies have shown amniotic membrane nerve wrap to significantly improve functional recovery, increase axon and fiber diameters, and increase myelin thickness when used around the sciatic^11^ and peroneal^12^ nerves.^9-12^ Because umbilical cord shares the same cell origin^13^ and has similar histologic features to amniotic membrane but possesses higher biological activity than amniotic membrane,^14^ we hypothesized that human umbilical cord allograft could be used as a nerve wrap during revision CTR to improve the recovery of median nerve function by reducing inflammation and preventing scarring. The purpose of this study was to evaluate the efficacy of human umbilical cord allograft to improve short- and long-term outcomes of revision CTR.

## METHODS

### Study Design

After approval from the institutional review board, we conducted a Health Insurance Portability and Accountability Act–compliant retrospective medical records review to identify patients who underwent revision CTR by a single surgeon with the adjunctive use of human umbilical cord allograft for persistent or recurrent compression neuropathy of the median nerve. The surgeries were performed between January 2015 and July 2018 at a single institution. The control patients underwent revision CTR without adjunctive use of human umbilical cord allograft between December 2011 and September 2015. Eligible patients were ≥18 years of age at the time of surgery and had recorded preoperative and postoperative subjective and objective findings regarding pain, paresthesia, and weakness mediated by the median nerve. Patients were excluded if they were lost to follow-up or if their most recent follow-up was <1 year postoperatively. Data collected from medical records included age, sex, time to initial revision, nerve conduction study (NCS) and electromyography (EMG) diagnosis, American Society of Anesthesiologists (ASA) physical status classification 2 and 3, most recent follow-up, postoperative pain medication use, last available Disabilities of the Arm, Shoulder and Hand (QuickDASH) score, ≤90-day complications, and >90-day CTR revisions.

### Surgical Technique

Open revision surgery was performed with the patient under regional anesthesia with a pneumatic tourniquet. An incision was made incorporating the previous incision using the Kaplan cardinal line distally and extending proximally across the wrist crease. The median nerve was identified proximally and distally with dissection down through the palmar incision under ×3.5 loop magnification. The status of the median nerve (color change, edema, laceration) and any evidence of incomplete release were recorded, and external neurolysis was performed to free the median nerve from the surrounding scar. No internal neurolysis was performed. Flexor retinaculum and transverse carpal ligament were ensured to be entirely released. No adjunctive treatment was used in the control group. Hemostasis was achieved after deflation of the tourniquet, followed by routine wound closure.

In the study group, after neurolysis, a 25 × 25-mm human cryopreserved umbilical cord allograft (Clarix Cord 1K, Amniox Medical, Inc.) was laid on the median nerve and sutured into the surrounding subcutaneous tissues—specifically into the released fascia that was overlying the ulnar nerve—with simple 4-0 Vicryl sutures at all 4 corners. In all but 3 study group patients, prior to placement of the umbilical cord allograft, a 10 × 40-mm porcine extracellular matrix nerve wrap was placed around the median nerve at the area of most compression which was defined by any of the following: flattening of the nerve secondary to chronic compression, discoloration, or hyperemia.

### Postoperative Care

Patients were placed in a volar slab wrist splint and routinely followed up in the office 2 weeks postoperatively to discontinue the splint and evaluate the incision site, range of motion, and degree of swelling. Patients were started on formal occupational therapy at that time and re-evaluated as needed at 6 weeks, 3 months, 6 months, and >1 year postoperatively. At their most recent follow-up, patients reported whether pain, paresthesia, and weakness were worse, the same, improved, or resolved. Physical examination findings were compared to preoperative findings for symptom resolution. The proportion of patients who had improvement or complete resolution of symptoms was compared between the study and control groups.

### Statistical Analysis

Differences in baseline characteristics were analyzed using Fisher exact test or chi-square test for categorical data and the Mann-Whitney *U* test for continuous data. Postoperative outcomes were analyzed using Fisher exact test for categorical data and *t* tests for continuous data. The level of statistical significance was set at *P*<0.05. Cohort size determination was based on primary outcome improvement incidence of 99.9% in the study group and 60% in the control group. To achieve 80% power (1−β) with α=0.05, enrollment of 22 patients in the study arm and 11 in the control arm was required to demonstrate statistical and clinical equivalence.

## RESULTS

A total of 37 patients (25 female, 12 male) with an average age of 65.3 ± 8.4 years (at most recent follow-up) were enrolled. All subjects underwent open revision CTR for recurrent or persistent compressive neuropathy with (n=26) and without (n=11) human umbilical cord allograft. No statistical differences were seen between the 2 groups regarding age, sex, time between initial CTR and revision surgery, and ASA physical status classification 2 or 3 (Table 1). Upon initial presentation, no statistical differences were seen in the 2 groups regarding NCS/EMG carpal tunnel syndrome grading as normal/negative, mild, moderate, or severe or the presenting symptoms of pain, paresthesia, or weakness.

**Table 1.** Demographics and Baseline Characteristics

Variable	Study Group, n=26	Control Group, n=11	*P* Value
Age at most recent follow-up, years			0.48
Mean (SD)	66.2 (8.4)	63.3 (8.2)	
Median (minimum, maximum)	67.0 (43, 84)	63.8 (47, 75)	
Sex			0.44
Female	19 (73)	6 (55)	
Male	7 (27)	5 (45)	
Time to revision, years			0.48
Mean (SD)	8.5 (6.5)	5.7 (4.6)	
Median (minimum, maximum)	7.0 (1.5, 21)	5.0 (0.25, 13)	
American Society of Anesthesiologists physical status classification			0.22
2	9 (35)	1 (9)	
3	17 (65)	10 (91)	
Nerve conduction study**/**electromyography carpal tunnel syndrome results			0.31
Normal/negative	2 (8)	1 (9)	
Mild	1 (4)	1 (9)	
Moderate	14 (54)	8 (73)	
Severe	9 (35)	1 (9)	
Presenting symptoms			0.99
Pain	25 (96)	11 (100)	
Paresthesia	25 (96)	11 (100)	
Weakness	19 (73)	9 (82)	

Notes: Data are presented as n (%) unless otherwise indicated. Carpal tunnel syndrome stratification by electrodiagnostic testing (nerve conduction study**/**electromyography): normal/negative—no abnormalities; mild—decreased sensory nerve conduction velocity, normal terminal motor latency; moderate—decreased sensory conduction velocity with preserved sensory potential, distal motor latency; severe—absent sensory potentials, distal motor latency to abductor pollicis brevis <6.5 ms.

The average follow-up period did not significantly differ between the groups, with 3.7 ± 1.1 years in the study group vs 4.5 ± 2.3 years in the control group (mean of 4.0 ± 1.6 years overall, *P*=0.16) (Table 2).

**Table 2.** Postoperative Outcomes

Variable	Study Group, n=26	Control Group, n=11	*P* Value
Time since revision, years			0.16
Mean (SD)	3.7 (1.1)	4.5 (2.3)	
Median (minimum, maximum)	3.9 (1.8, 5.5)	4.0 (1.0, 8.0)	
Symptomatic improvement or resolution			
Pain	24/25 (96)	8/11 (73)	0.048
Paresthesia	25/25 (100)	8/11 (73)	0.014
Weakness	19/21 (90)	6/9 (67)	0.14
QuickDASH score			0.58
Mean (SD)	19.0 (15.5)	23.7 (21.8)	
Median (minimum, maximum)	16.0 (0, 52.3)	22.7 (0, 63.6)	
Postoperative complications (≤90 days)	2/26 (8)	2/11 (18)	0.56
Carpal tunnel release revision (>90 days)	1/26 (4)	1/11 (9)	0.51
Time to pain medication cessation, weeks			0.99
Mean (SD)	2.9 (2.2)	2.9 (1.7)	
Median (minimum, maximum)	2.0 (2, 12)	2.0 (2, 6)	

Notes: Data are presented as n (%) unless otherwise indicated. Two patients in the study group and 1 patient in the control group did not complete postrevision QuickDASH surveys.

QuickDASH, Disabilities of the Arm, Shoulder and Hand.

Following open revision CTR, pain improved or entirely resolved in 96% (24/25) of patients with adjunctive umbilical cord vs 73% (8/11) of patients without (*P*=0.048). Paresthesia was improved or entirely resolved in 100% (25/25) of patients with adjunctive umbilical cord vs 73% (8/11) of patients without (*P*=0.014). Weakness was improved or entirely resolved in 90% (19/21) of patients with adjunctive umbilical cord versus 67% (6/9) of patients without (*P*=0.14). No patient in either group was noted to have incomplete release. Mean QuickDASH scores were improved but not significant in the study group (19.0 ± 15.5) vs the control group (23.7 ± 21.8, *P*=0.58). Two patients in the study group and 1 patient in the control group did not complete postrevision QuickDASH surveys.

Two patients in the study group (8%) and 2 patients in the control group (18%) had complications <90 days postoperatively (*P*=0.56). One study group patient had wound dehiscence and the other had recurrent symptoms that required neurolysis and scar revision 8 weeks postoperatively. Both control group patients presented with wound dehiscence, and 1 of these patients required antibiotics to treat surgical site infection. Only 1 patient in each group (2 total) required CTR revision >90 days postoperatively (*P*=0.51). Both patients developed recurrence of weakness and pain years after surgery that resolved after a third CTR revision. Time to pain medication cessation was nearly identical between the groups: 2.9 ± 2.2 weeks in the study group vs 2.9 ± 1.7 weeks in the control group (*P*=0.99).

## DISCUSSION

Primary carpal tunnel decompression is successful in the majority of patients. In patients with persistent or recurrent symptoms, anatomic findings at revision have demonstrated scar formation, incomplete release, proximal compression, swelling, compression from mass effect, and infection.^15^ Therefore, the goal of CTR revision for recurrent compressive neuropathy is to decompress the nerve, prevent recurrent scar formation, and promote functional recovery. However, revision decompression with or without neurolysis has less predictable and variable outcomes than primary decompression.^8,16^ In a study by Cobb et al, 11.5% of patients required a third procedure, and an additional 22.4% rated their outcome as not very successful (less than 50% relief) or completely unsuccessful.^5^ Our study showed that CTR revision with umbilical cord allograft achieved an overall success rate of 96% to 100% in relieving pain and paresthesia, which was significantly higher than 73% of the control group.

In an attempt to prevent scar formation and recurrent compression, other biomaterials have been used as a nerve wrap. Among them, hypothenar fat pad flaps are commonly favored given their proximity to the wound bed and availability of tissue,^7^ and they achieve consistent success ranging from 89% to 93%.^17^ Our study results support human umbilical cord as an alternative nerve wrap with the additional benefits of reducing operating time and donor site morbidity because umbilical cord allograft is commercially available. The clinical benefit observed in our study may be attributed to the anti-inflammatory and antiscarring effects of the umbilical cord allograft. The antiscarring effect is attributed to the high concentrations of heavy chain-hyaluronic acid/pentraxin 3 (HC-HA/PTX3) complex in the umbilical cord tissue. HC-HA/PTX3 complex has been demonstrated to suppress transforming growth factor beta 1 promoter activity, prevent myofibroblast differentiation, and inhibit expression of alpha smooth muscle actin.^18^ Moreover, these tissues have low immunogenicity and have been shown to promote nerve regeneration after repair of transected sciatic nerves, perhaps because of the rich composition of growth factors, proteins, and neurotrophic factors.^19^ Collectively, these actions may promote a favorable microenvironment to correct the pathology inherent in recurrent compression neuropathy as illustrated in our study and reported in preclinical studies^9-12^ and a case series^20^ in which amnion nerve wrapping was used to treat entrapment of the superficial sensory branch of the radial nerve. Therefore, human umbilical cord allograft may be an alternative nerve graft to address recurrent and refractory compressive neuropathy in patients, especially those with risk factors such as female sex, age 40 to 49 years, diabetes, hypertension, obesity, and hand osteoarthritis, as well as occupational groups that perform repetitive hand motions.^6,21,22^ The preliminary encouraging results from our study warrant further validation by prospective randomized trials.

Our study has limitations. Additional patients would have increased the power of the study. Future studies should aim to increase the sample size and demonstrate consistency of the results of CTR with human umbilical cord allograft. The retrospective design of this study is another limitation. With a prospective study, we would likely obtain better subjective and objective evaluations of median nerve compression, including quantification of numbness, tingling, pain, and other symptoms. A third limitation is that we used both human umbilical cord allograft and porcine extracellular matrix nerve wrap in our study group compared to no allograft in the control group. Benefit could potentially be derived from either biomaterial. Studies are needed to further elucidate. With an average age of 65 years, many of the patients in our study may have had degenerative changes of the cervical spine and upper extremity, leading to less specificity of the QuickDASH score for median nerve symptoms and therefore less pronounced differences between the 2 groups. In future controlled trials, a more specific median nerve compression measurement tool should be used.

## CONCLUSION

This study demonstrates the safety and efficacy of using adjunctive human umbilical cord allograft to improve the long-term outcomes of patients with recurrent, persistent, and refractory compressive neuropathy resulting from carpal tunnel syndrome.

## Notes

*This article meets the Accreditation Council for Graduate Medical Education and the American Board of Medical Specialties Maintenance of Certification competencies for Patient Care, Medical Knowledge, and Practice-Based Learning and Improvement.*
